# Impact of Maternal Diet on the Epigenome during *In Utero* Life and the Developmental Programming of Diseases in Childhood and Adulthood

**DOI:** 10.3390/nu7115467

**Published:** 2015-11-16

**Authors:** Ho-Sun Lee

**Affiliations:** Epigenetics Group, International Agency for Research on Cancer (IARC), 150 Cours Albert-Thomas, 69372 Cedex 08, France; hs4369@gmail.com; Tel.: +33-4-7273-8398

**Keywords:** epigenetics, development, maternal nutrients, metabolic syndrome

## Abstract

Exposure to environmental factors in early life can influence developmental processes and long-term health in humans. Early life nutrition and maternal diet are well-known examples of conditions shown to influence the risk of developing metabolic diseases, including type 2 diabetes mellitus and cardiovascular diseases, in adulthood. It is increasingly accepted that environmental compounds, including nutrients, can produce changes in the genome activity that, in spite of not altering the DNA sequence, can produce important, stable and, in some instances, transgenerational alterations in the phenotype. Epigenetics refers to changes in gene function that cannot be explained by changes in the DNA sequence, with DNA methylation patterns/histone modifications that can make important contributions to epigenetic memory. The epigenome can be considered as an interface between the genome and the environment that is central to the generation of phenotypes and their stability throughout the life course. To better understand the role of maternal health and nutrition in the initiation and progression of diseases in childhood and adulthood, it is necessary to identify the physiological and/or pathological roles of specific nutrients on the epigenome and how dietary interventions *in utero* and early life could modulate disease risk through epigenomic alteration.

## 1. Introduction

Maternal diet plays a critical role in fetal growth and development. Pregnancy is associated with increased nutritional needs due to the physiological changes of the mother and the metabolic demands of the embryo/fetus (conceptus). Therefore, the conceptus requires increased calories, macronutrients and micronutrients, for growth and development [1]. A growing number of studies focusing on the developmental origin of health and disease have identified links between early nutrition and disease, consistent with the “developmental origins of health and disease” (DOHaD) hypothesis [2]. This hypothesis postulates that early life development is critically sensitive to inadequate nutrition and other environmental factors, leading to permanent changes in development that can influence the health of an individual in later life and the risk of disease [2,3].

Although causal relationships have not been firmly established, recent advances in epigenetics provide insights into the mechanisms of early life predisposition to adult disease risk. Should some of the epigenetic mechanisms underlie developmental plasticity in the DOHaD and disease-related outcomes result from disruptions of the epigenome induced by the fetal environment, this emerging field may provide critical insights into the role of early life events on disease programming in childhood and adulthood [4]. Epigenetic modifications are stable during cell division, and they regulate gene activity states over cell generations [5]. During development, epigenetic marks, such as DNA methylation, histone modification and microRNA expression, undergo substantial reconfiguration, which affects the genes and pathways that are essential for both early life development and later life physiological functions. Experimental studies supported the notion that prenatal nutritional constraints may induce an altered metabolic phenotype in the offspring, which constitute an increased risk of non-communicable diseases, including obesity, diabetes and cardiovascular diseases, in humans [6,7]. However, the precise mechanisms involved have not been fully elucidated.

Here, we review recent evidence on the potential of maternal diet *in utero* to counteract epigenetic alterations and phenotypic outcomes and discuss its implications on novel strategies for epigenetic chemoprevention during pregnancy.

## 2. Epigenetic Mechanisms

Epigenetic markers are enzyme-mediated chemical modifications of DNA and of its associated chromatin proteins. These modifications play key roles in regulating genomic functions, without altering the primary DNA sequence, and are transmitted with high fidelity over many cell generations. Such marks influence the transcriptional state and other functional aspects of chromatin. For examples, methylation of DNA and certain residues on the histone H3 N-terminal tail are important for transcriptional gene silencing and the formation of heterochromatin. The main epigenetic mechanisms are DNA methylation, histone modifications and microRNA, which are involved in the propagation of the chromatic structure and genome activity states.

### 2.1. DNA Methylation

DNA methylation is the most extensively-studied epigenetic modification that is mediated by a group of dedicated enzymes. Changing DNA methylation patterns in key elements of the gene, such as promoters and enhancers, can have a profound effect on gene function. The best characterized examples of methylation-mediated silencing mechanisms are genomic imprinting, X-chromosome inactivation and silencing of retrotransposons [8,9]. Imprinted genes specifically have one or more transcripts that are expressed preferentially or exclusively from one parental allele. This monoallelic expression is likely to be initiated by differential methylation between the oocyte and sperm in differentially-methylated regions (DMRs) at gametes. Soon after fertilization (periconceptional period), this differential methylation leads to additional molecular changes, including allele-specific methylation at somatic DMRs, which ultimately result in expression exclusively from one allele. It is perhaps during these periods of development, then the epigenome undergoing physical depletion, followed by renewal and being in a highly dynamic state of flux, that any endogenous or exogenous influences may be most likely to induce persistent changes in methylation. Maintenance of DNA methylation is mediated by DNA methyltransferases (DNMTs), and this process is relatively well understood at a biochemical level. In contrast, much remains to be learned about the establishment of DNA methylation (*de novo* methylation) and the mechanisms underlying its deregulation. In contrast, much remains to be learned about the establishment of DNA methylation (*de novo* methylation) and the mechanisms underlying its deregulation. Demethylation of the genome occurs during two developmental periods; during preimplantation development and in primordial germ cells (PGCs). As these mechanisms operate in strictly-defined stages of development, it is possible that these developmental periods (such as the peri-conceptual period) represent particularly sensitive windows of vulnerability. Rapid DNA demethylation of the paternal pronucleus occurs post-fertilization in the zygote, but before cell division and at least partly before DNA replication. This contrasts with the gradual loss of methylation from the maternal genome through the first few cleavage divisions in the embryos. After implantation, global *de novo* methylation is set up and maintained in somatic tissues. Concerning DNA demethylation mechanism in mammalian genomes, the 5mC hydroxylases TET1 and TET2 are also expressed in PGCs, suggesting the possibility that 5mC could be modified by different mechanisms (e.g., formylation, carboxylation, hydroxylation) in order to initiate active demethylation [10,11]. Thymine DNA glycosylase was also shown to be involved in these mechanisms [12]. Recently, *tet3* was shown to be required for paternal and maternal DNA demethylation coupled to DNA replication [13].

DNA methylation is dependent on the one-carbon metabolism pathway, which depends on different enzymes, whose activities, in turn, rely on micronutrients supplied through the diet. Through an ATP-driven reaction, methionine is converted into *S*-adenosylmethionine (SAM), the universal cellular methyl donor. Methyl-tetrahydrofolate (THF) acts as a methyl-group donor for the production of THF and is a precursor for homocysteine conversion to methionine. DNMTs covalently attach methyl groups from SAM to the carbon-5 position of cytosine bases, generating 5mC. Different nutrients involved in one-carbon metabolism, such as folate, vitamin B6, vitamin B12, choline and methionine, play an important role in DNA methylation through their influence on SAM levels and methyltransferase inhibitor S-adenosylhomocysteine (SAH) levels. Other nutrients and bioactive food components, such as retinoic acid, resveratrol, curcumin, sulforaphane and tea polyphenols, can modulate epigenetic patterns by altering SAM and SAH levels or regulating DNA methylation and histone modifications in specific genes or at the genome-wide level. Some of these nutrients are currently evaluated as optimal maternal nutrition to modulate epigenetic mechanisms [14].

### 2.2. Histone Modification

Histones are basic proteins that facilitate the packaging of DNA in the nucleus and the regulation of gene expression on cells. Nucleosomes are composed of 147 bp of DNA wrapped around an octamer of core histone proteins, namely H2A, H2B, H3 and H4. Epigenetic regulation of gene expression is also mediated by post-translational modifications, including acetylation, methylation, phosphorylation, ubiquitinylation and SUMOylation, at the N-terminal tails of histones, thus contributing to genomic stability, DNA damage response and cell cycle checkpoint integrity. Modifications in histone methylation are different between cell types and are associated with fetal development. Methylation of histone tails is not only restricted to lysines, but can also occur at arginine residues on the histone-H3 and -H4 tails (H3R2, H3R8, H3R17, H3R26 and H4R3). Several protein arginine methyltransferases (PRMTs) have been associated with epigenetic regulation, in which PRMTs act as histone methyltransferases or secondary co-regulators of transcription, or facilitate mRNA splicing and stability. There are eleven known PRMTs (PRMT1–PRMT11) that have important roles in chromatin modeling and transcriptional states [15]. Among these, PRMT1 catalyzes the addition of two methyl groups to the same terminal guanidino-nitrogen group of arginine in proteins, resulting in methylation of *PGC1-*α, which then relays changes in fatty acid oxidation and energy metabolism [16]. Histone methylation can also be reversed by histone demethylases. The histone demethylase, *jhdm2a*, for example, was identified as a crucial regulator of the genes involved in energy expenditure and fat storage, suggesting that this enzyme may be a key factor in obesity and metabolic syndromes [17]. Histone acetylation is maintained by the interplay of histone acetyltransferases (HATs) and histone deacetylases (HDACs) [18]. Acetylation of lysine residues at the N-terminus of histone proteins is catalyzed by HATs, including *GNAT*, *CBP/p300* and *MYST*. Notably, acetylation of lysine residues neutralizes the positive charge on histones, thereby decreasing the interaction of histone N-termini with the negatively-charged phosphate groups of DNA. Such histone modifications allow easier access to transcription factors [18]. Histone deacetylation is catalyzed by HDAC with the acetyl group transferred from the acetylated histone protein to coenzyme A. Recently, a new mechanism suggested that glucose metabolism is linked to chromatin modification and global transcriptional control via the enzyme ATP-citrate lyase and production of acetyl coenzyme A, hence providing additional evidence for a glucose-to-gene link [17]. From this consideration, histone modification may be one of the key mechanisms for controlling adipogenesis and energy homeostasis.

### 2.3. MicroRNA

The human genome contains only 20,000 protein-coding genes, representing <2% of the total genome, whereas a substantial fraction of the human genome can be transcribed, yielding many short- or noncoding-RNAs (ncRNAs) with limited protein-coding capacity. Among these, the most extensively-studied ncRNAs are microRNAs (miRNAs, 20–22 nt), which are evolutionarily conserved and located within the introns and exons of protein-coding genes (70%) or in intergenic regions (30%). Dietary factors have been shown to modify miRNA expression profiling, notably in maternal nutrition-induced epigenetic modifications in offspring with lipid metabolism, insulin resistance and inflammation [19,20,21]. However, the role of miRNAs in fetal programming remains largely understudied.

## 3. The Role of Epigenetics in Early Development and Disease

Epigenetic marks are critical for cellular differentiation during development and the differentiation of individual tissues [22]. Epigenetic reprogramming during embryo development induces the most extensive changes to the epigenetic state. The epigenetic state that is set during the reprogramming stages persists at some loci for the lifetime of an individual. Evidence to support a role of epigenetics in developmental programming of disease demonstrated the impact of a suboptimal intrauterine nutritional environment on the epigenome and phenotype of the offspring. For example, nutritional influences on developmental epigenetics were first suggested by studies on agouti mice with the *viable yellow agouti* (*A^vy^*) metastable epiallele (ME), associated with contraoriented intracisternal A particle proviral insertions, which encodes a paracrine signaling molecule. It promotes follicular melanocytes to produce a yellow-rather than black-colored coat, obesity, diabetes and increased susceptibility to tumors. Early life exposures can be “stored” in the cellular memory by inducing lasting changes in gene expression programs and molecular pathways with long-term effects. Consequently, alterations in DNA methylation and histone markers eventually contribute to the development of age-related and lifestyle-related diseases, such as metabolic disease, cardiovascular disease, asthma, neurological disorders, like Alzheimer’s disease, and cancer [1,23,24]. However, details remain largely undefined in human studies.

## 4. The Impact of Maternal Nutrition on Fetal Programming in Animal Models

Epigenetic effects of maternal nutrition on offspring during early development have been demonstrated using several animal models, including *A^vy^* and axin-fused (*Axin^Fu^*) mice. Supplementation of methyl donors including folate during pregnancy increased DNA methylation of the agouti gene in *A^vy^* offspring , leading to low agouti expression and yellow to brown coat color [8]. *Axin^Fu^* also exhibited epigenetic plasticity to maternal diet. Methyl donor supplementation of female mice during pregnancy induced DNA hypermethylation at *Axin^Fu^* and, thereby, reduced by half the incidence of kinking in *Axin^Fu^/+* offspring [25]. A number of manipulations have also been used to induce offspring intrauterine growth retardation (IUGR) in animal models, including maternal calorie and protein restriction via epigenetic mechanisms [26,27]. Animal models have been used in an attempt to understand the molecular basis of the relationship between maternal dietary supplementation/deficiency and the development of adult diseases, like metabolic syndrome (Table 1).

**Table 1:** Effect of maternal nutritional exposure *in utero* on epigenetic modifications in animal studies.

Nutrients	Mechanism	Outcome	Model	Target	Ref.
Methyl donors ^(1)^	DNA methylation	↓↑ methylation of genes in rennin-angiotensin system	rat	liver	[28]
	Histone modification	Sirt1 and pmrt1 expression ↓, PGC-1α acetylation ↑	rat	heart	[29]
	DNA methylation	↓ methylation of PGC-1α	rat	liver	[29]
Methyl donors ^(^^2)^	DNA methylation	↓ Methylation of *Ptpn22* and *Ppara*	mouse	mucosa	[30]
Choline	DNA methylation	↓ methylation of *Vegfc* and *Angpt2* promoter	mouse	brain	[31]
		Global methylation ↓			
	DNA methylation	↓ methylation of *Cdkn3* promoter	mouse	brain	[32]
	Histone modification ^(3)^	H3K9me2 (active) and H3K27me3(repression) ↓	rat	liver	[33]
		H3K4me2 ↑ G9a and Suv39h1 (HMT) expression ↓		brain	
	Histone modification ^(4)^	H3K9me2 (active) and H3K27me3(repression) ↑	rat	liver	[33]
		H3K4me2 ↓; G9a and Suv39h1(HMT) expression ↑		brain	
	DNA methylation	↑ methylation of *IGF2*, Dnmt1 expression ↓	rat	liver	[34]
	DNA methylation	Global methylation ↑	rat	liver	[35]
undernutrition	DNA methylation	↓ methylation of *IGF2/h19*	sheep	blood	[36]
Overfeeding	DNA methylation	↑methylation of exon 3 in *Pomc*	mice	blood	[37]
	DNA methylation	↑ methylation within Sp1-related binding sequences	rat	brain	[38]
High fat	Histone modification	↑ acetylation of H3K9, H3K18 and H3K14	macaque	liver	[39]
	DNA methylation	↓ methylation of dopamine reuptake transporter and	mouse	brain	[40]
		μ-opioid receptor and preproenkephalin promoter			
	Histone modification	↓SIRT expression, ↑ acetylation of H3K14	macaque	liver	[41]
	Histone modification	↓SIRT expression	rat	heart	[42]
undernutrition	DNA methylation	↓ methylation of DMRs	mouse	sperm	[43]
	DNA methylation	↓↑methylation of 5′ UTR region	mouse	liver	[44]
Protein	DNA methylation	↑ methylation of *Wnt* promoter	rat	placenta	[45]
	DNA methylation	*Agtr1b* promoter	rat	kidney	[46]
	Histone modification	↓ Dnmt1 expression, ↓ methylation of *GR* 1 _10_ promoter	rat	liver	[47]
	Histone modification	Interaction between *Hnf4a* enhancer and *p2* promoter	rat	liver	[48]
				pancreatic islets	
	DNA methylation	↑ methylation of *IGF2/H19* ICR	rat	liver	[49]
	Histone modification	Dmnt1,3a and *Mbd2* expression ↑			
	DNA methylation	↑ methylation of *X-receptor* promoter	mouse	liver	[50]
Alcohol	Histone/Protein modification	↑ acetylation of *PTEN* and TRB3, HAT activity ↑	rat	liver	[51]
	DNA methylation	↑ methylation of A^*vy*^ allele,	mouse	blood	[52]
	DNA methylation	Global hypomethylation	mouse	blood	[53]
	DNA methylation	↓ CTCF-binding site 2 of *h19*	mouse	sperm	[54]
	Histone modification	↑ HDAC (4,5,7) and SIRT2 protein level	rat	liver	[55]
		↑ acetyl-foxo1 protein level			

^(1)^ Folate, vitamin B12; ^(2)^ folate, vit B12, betaine and choline; ^(3)^ by choline deficiency; ^(4)^ by choline supplementation; *Vegfc*, VEGF-C; Angpt2, angiopoietin 2; *Ppar* α, peroxisomal proliferator-activated receptor α; Agtr1b, angiotensin II receptor, type 1b; *Hnf4a*, hepatocyte nuclear factor 4a; Cdkn3, cyclin-dependent kinase; GR, glucocorticoid receptor; *A^vy^*, *agouti viable yellow*; DMR, differentially methylated region.

### 4.1. One-Carbon Metabolism-Related Nutrients

Methyl group metabolism has emerged as a metabolic process that has profound effects on diseases [56]. Methyl deficiency and the compensatory increase in Hcy are a significant risk factor for cardiovascular disease [57]. For example, impaired activity of one-carbon metabolism enzymes may lead to an aberrant DNA methylation pattern and increased plasma Hcy, which is a toxic derivative that leads to vascular lesions. In addition, it was reported that restriction of folate, methionine and B vitamins during the periconceptional period resulted in altered DNA methylation, insulin resistance and elevated blood pressure observed in adult male offspring [58], as well as gene expression and methylation changes involved in the renin-angiotensin system, mitochondrion metabolism and phospholipid homeostasis [28]. Additionally, deficiencies in folate and vitamin B12 during gestation and lactation produced manifestations of fetal programming, with decreased birth weight, increased central fat mass, liver steatosis and myocardium hypertrophy in pups from deficient rat mothers [16]. The underlying molecular mechanisms are linked to the decreased expression and activity of *SIRT1* and *PRMT1* and the subsequent hyperacetylation and hypomethylation of *PGC1-*α [29]. However, methyl donor supplementation in utero also enhanced colitis susceptibility, which was associated with aberrant DNA methylation among genes associated with immunologic processes [30].

Choline may have important roles in fetal brain development, as maternal choline deficiency during pregnancy has been shown to modify the epigenome of the fetal brain. For example, *Vegfc* and *Angpt2* [31], which are involved in angiogenesis signaling, and *Cdkn3* [32], for cell proliferation, were hypomethylated in the fetal brain. Additionally, levels of H3K9me2 and H3K27Me3 (repressed) were upregulated in the fetal liver and brain by maternal choline supplementation, whereas levels of H3K4me2 (active) were highest in choline-deficient rats. This was further supported by experiments showing that H3K9me2 levels were correlated inversely with the induction of peroxisomal proliferator-activated receptor γ (PPARγ) in both murine and human adipogenesis [33]. Prenatal choline deficiency also altered imprinted genes’, like *IGF2*, methylation and expression through effects on Dnmt1 in liver [34]. However, choline supply has also been shown to modify the DNA methylation and expression of genes involved in methionine and lipid metabolism in Wilson disease [35]. Therefore, the nutrients regulating maternal-fetal one-carbon metabolism may play important roles in the understanding of fetal programming and metabolic diseases.

### 4.2. Under-/Over-nutrition

Epidemiological studies have linked the impact of maternal nutrition with the etiology of chronic diseases in offspring in their adult life. Nutritional imbalances, such as under- and over-nutrition during critical periods, induce persistent physiological alterations. Maternal undernutrition during early pregnancy, in relation to a low birth weight and uterine growth restriction, may adversely influence offspring metabolism and health. There are several human and animal studies linking suboptimal early nutrition and poor growth *in utero* with increased risk of coronary disease, hypertension, type 2 diabetes and obesity in adulthood [59,60]. Some studies suggested that maternal undernutrition may accelerate early postnatal growth by an increased rate of gaining body fat rather than muscle tissue, with the implication that this was triggered by catch-up growth on insulin resistance [1,61]. Researchers investigated the biological process associated with this phenomenon and identified that epigenetic mechanisms can be responsible for this fetal programming. One of the most significant studies was conducted in children who were born to women exposed to severe undernutrition during pregnancy as a result of the Dutch Hunger Winter during World War II. This study reported a reduced methylation of the imprinted gene *IGF2* in these individuals in adulthood [62]. Similarly, maternal undernutrition was associated with an increased risk of adult-onset metabolic syndrome, and the most consistently-observed epigenetic association with adiposity has been with that of methylation at the *IGF2/H19* imprinting region [36,63]. Higher methylation levels at specific genes, including *IL10*, *LEP*, *ABCA1*, *GNASAS* and *MEG3*, were closely linked with nutrition metabolism, cardiovascular function and inflammation [64]. Moreover, alteration in hypothalamic cell proliferation and impaired hypothalamic response have been observed in perinatally-undernourished offspring [65].

With the recent rise in childhood obesity, maternal obesity and excessive gestational weight gain are now considered risk factors of many adverse offspring short- and long-term outcomes through epigenetic mechanisms [66]. Several studies have assessed the relationship between DNA methylation in obesity markers, including *RXR*α and *NOS3*, in umbilical cord blood cells with childhood adiposity [67], pre-proopiomelanocortin (POMC) in peripheral blood cells with an increased individual risk for the development of obesity [37], melanin-concentrating hormone receptor 1 (*MCHR1*) in peripheral blood cells [68] and the androgen receptor (AR) promoter in peripheral blood leukocytes with body mass index (BMI) or fat mass [69]. These studies provided evidence that obesity is associated with altered epigenetic regulation of a number of metabolically-important genes. Maternal over-nutrition is also related to the origin of other metabolic diseases, such as hypertension and hyperlipidemia or insulin resistance and diabetes in offspring [70,71]. Maternal high fat diet (HFD) is associated with changes in hypothalamic regulation of body weight and energy homeostasis by altering the expression of leptin receptor, *POMC* and *neuropeptide Y* in offspring [72]. The mechanisms affected by nutritional challenges may alter permanent dysregulation of these hypothalamic circuits, including functional resistance to insulin and leptin, which may underlie permanently increased food intake and overweight status [38]. Further examples include global hyper-acetylation of histone H3 in the offspring [39] and alteration of methylation and expression of genes related to the mesocorticolimbic reward circuitry (dopamine and opioids) by maternal HFD [40]. *SIRT1*, a member of the HDAC family, promotes fat metabolism in adipocytes by repressing PPARγ, which means that upregulating *SIRT1* increases lipolysis, thereby inducing fat loss. Additionally, *SIRT1* is involved in the regulation of glucose homeostasis, insulin sensitivity, oxidative stress and anti-inflammatory activity [73,74,75]. Recent data have shown that maternal HFD leads to metabolic programming through increasing the acetylation of histone H3K14 and decreasing *SIRT1* expression in fetal liver [41] and heart [42]. Therefore, SIRT1 is supposed to be an epigenetic target of the fetal development for susceptibility to metabolic outcomes.

A number of studies for the transgenerational effect of metabolic diseases have shown the effects of maternal perinatal under-/over-nutrition, even though the mechanisms underlying transgenerational effects of developing programming remain poorly understood. Maternal nutrition imbalance was shown to exhibit transgenerational effects through epigenetic and metabolic changes [76,77]. Recently, maternal malnutrition induced germline DNA methylation in sperms of the first generation in the intrauterine condition, which altered the metabolic phenotype in the second generation [43,44]. Therefore, maternal nutrition state in early life modulates “epigenetic programming” that modifies signals, which continues in later life in lipid metabolism, appetite, the reward pathway in the brain and inflammation, even via transgenerational transmission.

### 4.3. Protein Deficiency

The metabolism of amino acids, such as glycine, histidine, methionine and serine, plays a key role in supplying methyl donors for DNA and protein synthesis [78]. For example, IUGR rat model’s under gestational protein deficiency showed site-specific hypomethylation of the *Wnt2* promoter in the placenta [45]. These findings were supported by the fact that *Wnt2* promoter methylation in the placenta is associated with fetal growth. A low protein (LP) diet is associated with impaired fetal growth and the development of obesity, diabetes and hypertension in the offspring [79]; for example, prenatal protein restriction changed the amount of DNA methylation and expression of the *agtr1b* gene, which is implicated in hypertension [46]. LP maternal diet has also been reported to influence promoter methylation status and expression of the *GR* and *PPAR* genes in liver of mice offspring [47] via acetylation of histone H3 and H4 and methylation of H3K4. Both GR and PPARs are important for normal embryogenesis and regulation of blood pressure or lipids in adults [80,81]. These findings were supported by observations that insulin secretion is regulated by histone modifications. Hyperacetylation of H4 and hypermethylation of H3K4 play important roles in the β-cell-specific expression of the insulin gene [82]. Another example is the incidence of type 2 diabetes in rats, for which LP diets were associated with the epigenetic repression of *hepatocyte nuclear factor 4a (Hnf4a)* in pancreatic islets. This gene encodes a transcription factor involved in β-cell differentiation and glucose homeostasis [48], as well as decreased *cholesterol 7*α*-hydroxylase (Cyp7a1)* expression. LP diet during fetal development resulted in DNA hypermethylation of the *H19/IGF2* locus with increased expressions of DNMT1, DNMT3a and methyl CpG binding domain protein 2 in liver of male offspring [49]. Additionally, LP diet was associated with DNA hypermethylation of the liver *X-receptor (LXR)*α promoter involved in glucose homeostasis and its target genes *Abcg5/Abcg8* in fetal liver [50]. Similarly, an increase in mRNA and protein expression of the CCAAT/enhancer-binding protein (C/EBPb), a transcription factor that regulates the expression of genes involved in energy homeostasis, was found to be altered in LP diet [58].

### 4.4. Alcohol Exposure

We include maternal alcohol intake in this review, given that alcohol interferes with nutritional supply to the fetal-placental unit and that a nutritionally inadequate maternal diet has been demonstrated to exacerbate the effects of ethanol [83]. Recent animal studies have demonstrated that prenatal ethanol exposure causes deficits in metabolic pathways in offspring, manifesting as increased whole-body adiposity, altered pancreatic beta cell structure, impaired glucose metabolism and insulin resistance [51,84]. A direct link exists between ethanol exposure and aberrations in DNA methylation [52]. Excessive alcohol is known to alter normal folate metabolism and to reduce its bioavailability. It was reported that alcohol-fed rats displayed reduced methionine synthase activity and low levels of both methionine and SAM [85]. Additionally, ethanol appears to enhance the loss of methyl groups, which, in turn, disrupts subsequent SAM-dependent transmethylation reactions [86]. For example, evaluating the effects of *in utero* ethanol exposure from Days 9–11 of gestation, acute ethanol administration to pregnant mice resulted in lower-than-normal methylation throughout the genome (*i.e.*, in global hypomethylation) of fetal DNA [53]. Furthermore, the ethanol-exposed fetuses displayed significantly reduced levels of DNA methylase activity. Ethanol-induced reductions in DNA methylation affected not only the fetus, but also the placenta of pregnant mice [87]. More recently, researchers found that prenatal alcohol exposure was involved in the gene expression and methylation status of imprinted genes in the sperm of male offspring [54], a finding also observed in heavy alcohol drinkers [88]. Additional studies assessed the effects of alcohol on histone modifications. Prenatal alcohol exposure resulted in increases of histone deacetylase and the reduction of acetylation in *foxo1* and PEPCK with glucose intolerance [55]. Yao *et al.* also reported that hepatic insulin resistance induced by alcohol exposure was associated with reduced acetylation of the TRB3 and PTEN proteins [51]. However, establishing a causality between prenatal alcohol exposure and epigenetic changes is still a challenge.

## 5. Human Studies with Diet and Its Impact on Epigenomics

Persistent epigenetic differences induced by environmental exposures may underlie such epidemiological observations. The Dutch hunger study on individuals conceived during the famine suggested that this famine was associated with different adverse metabolic phenotypes, such as higher BMI, elevated serum cholesterol or impaired glucose tolerance and increased risk of insulin resistance, depending on the phase of development at exposure [62]. However, such findings were difficult to replicate in other studies. In the Finnish famine cohort, individuals experiencing nutritional deprivation *in utero* exhibited a rise in mortality in early life, but went on to have a lifespan not significantly different from that of non-exposed individuals [89]. In a recent study on children born to mothers exposed to aflatoxin in Sub-Saharan Africa, maternal exposure to aflatoxin during the early stages of pregnancy was found to be associated with changes in the DNA methylation patterns of the infants [90], reinforcing the notion that exposure during critical periods of fetal development may have an impact on the epigenome and infant development

The importance of one-carbon metabolism in fetal programming was reported through evidence indicating that low maternal vitamin B12 levels correlate strongly with hyperhomocysteine levels and predicted higher offspring adiposity and higher insulin resistance [91,92] in humans. Global methylation and imprinted gene methylation were also modified by one-carbon metabolism [93,94]. Among the nutrients involved in one-carbon metabolism, folic acid has been studied widely and is implemented universally for disease prevention (Table 2). In particular, the methylation of imprinted genes like *IGF2/H19* DMR was well studied with maternal folate supplementation with ≥400 µg/day [95,96,97]. For the effect of choline supplementation, a higher maternal choline intake (930 *vs*. 480 mg/day) increased placental *CRH* and *GR* methylation and decreased placental *CRH* transcript abundance, indicating that extra choline during pregnancy decreased placental expression of cortisol-regulating genes [98]. Our group also reported that DHA supplementation with 400 mg/day during pregnancy induced DNA methylation changes in inflammatory genes and *LINE-1*, especially in infants born to mothers who smoked during pregnancy [99].

**Table 2:** Intervention studies for maternal nutritional status and epigenetic profile in offspring.

Nutrients	Mechanism	Outcome	Sample	Method	Ref.
Folate and Multivitamins ^(1)^	DNA methylation	↓ methylation of *IGF2 2R* in girls, ↓ methylation of *GTL2-2* in boys	Cord blood	27k Illumina	[93]
Folate	DNA methylation	↓ methylation of *LINE1*	Cord blood	Pyrosequencing	[94]
	DNA methylation	↑ methylation of IGF2 DMR	Cord blood	Epi-typer	[95]
	DNA methylation	↓ methylation of H19 DMR (4 CpGs)	Cord blood	Pyrosequencing	[96]
	DNA methylation	↓ Methylation of *PEG3* and *LINE1*; ↑ methylation of *IGF2*	Cord blood	Pyrosequencing	[97]
Choline	DNA methylation Histone modification	↑ methylation of *CRH*, *NR3C1* in placenta; ↓ methylation of *CRH*, *NR3C1* in cord blood leukocyte; ↑ H3K9me2	Placenta, cord blood	LC-MS/MS	[98]
DHA	DNA methylation	↑ *LINE1* methylation ↑ *INFγ/IL13* methylation ratio	Cord blood	Pyrosequencing	[99]

^(1)^ Zinc and vitamins A, B, C and D; PEG3, paternally-expressed gene 3; CRH, corticotropin-releasing hormone; NR3C1, nuclear receptor subfamily 3, group C, member 1.

Metastable epialleles are characterized by stochastic exposure and are more likely to be affected by environmental exposure. Waterland *et al.* used genome-wide methylation-specific analysis for ME screening to identify differences in DNA methylation based on the season of conception (dry/rainy) in rural Gambia. Distinct loci during the rainy season resulted in significantly higher DNA methylation, providing evidence suggesting that environmental changes also affected ME [100,101]. Similarly, Cooper *et al.* reported sex-specific effects of micronutrient supplementation, with reduced methylation levels at two of the DMRs in imprinted genes, IGF2R in girls and GTL2-2 in boys [93]. High levels of animal proteins and low-carbohydrate diet during pregnancy have been shown to be associated with increases in blood pressure and cortisol in response to stress in adults [102]. Drake *et al.* reported that maternal unbalanced diet (rich in proteins and low in carbohydrates) in early life correlates with methylation at specific candidate loci some 40 years later in the offspring. The authors also found changes in methylations within GR, 11β-hydroxysteroid dehydrogenase type 2 and *IGF2*, which were positively associated with increased adiposity and blood pressure in adults [103].

Not only maternal, but also paternal diet may regulate epigenetic modification in the offspring. The Swedish Overkalix cohort study showed that food supply in the adolescence of paternal grandparents correlated with the grandchild’s longevity and susceptibility to the risk of cardiovascular death or diabetic disease. In a Dutch famine study, the offspring of prenatally-undernourished fathers, but not mothers, was heavier and more obese than that of fathers and mothers who had not been undernourished prenatally [104]. The epigenetic gametic inheritance may be a possible explanation indicating the effects of paternal nutrition on developmental programming, which might be a transgenerational phenomenon and can be viewed as a form of epigenetic inheritance [105]. However, such evidence is still lacking in humans.

## 6. Summary and Conclusions

Distinct programs of gene expression underlying the development of different cell lineages and tissues are executed (with minor exceptions) without changes in the genome (DNA sequence). Epigenetic reprogramming is essential during embryonic and early postnatal development, and epigenetic deregulation is recognized to play a role in the etiology of several developmental syndromes. Current literature clearly shows that the effects of various *in utero* exposures and maternal nutritional status may have different effects on the epigenome. However, critical windows of exposure that seem to exist during development need to be better defined. Although it is widely agreed that suboptimal maternal nutrition has a negative impact on the health of the offspring, effective intervention strategies aiming to modulate the epigenome and to prevent the development of complex human diseases, as a consequence of nutritional programming, is still challenging. To obtain a better understanding of the role of maternal health and nutrition in the initiation and progression of metabolic and other disorders, it is necessary to identify the common mechanisms and pathways involved in disparate perinatal malnutrition paradigms in order to decipher the physiological and/or pathological roles of specific nutrients.

## Abbreviations

DOHaD: developmental origins of health and disease; DMRs: differentially-methylated regions; DNMTs: DNA methyltransferases; PGCs: primordial germ cells; SAM: S-adenosylmethionine; SAH: S-adenosylhomocysteine; HMTs: histone methyltransferases; HATs: histone acetyltransferases; HDACs: histone deacetylases; Avy: viable yellow agouti; Angp2: angioprotein 2; PPARγ: peroxisomal proliferator-activated receptor γ; LP: low protein; Cdkn3: cyclin-dependent kinases 3; GR: glucocorticoid receptor; Hnf4a: hepatocyte nuclear factor 4a; Cyp7a1: cholesterol 7α-hydroxylase; LXR: X-receptor; C/EBPb: CCAAT/enhancer-binding protein; POMC: pre-proopiomelanocortin; MCHR1: hormone receptor 1; HFD: maternal high fat diet; CEP: CREB binding protein; MEs: metastable epialleles; PEG3: paternally-expressed gene 3; CRH: corticotropin releasing hormone; NR3C1: nuclear receptor subfamily 3, group C, member 1.
